# From Online Randomized Controlled Trials to Participant Preference Studies: Morphing the San Francisco Stop Smoking Site into a Worldwide Smoking Cessation Resource

**DOI:** 10.2196/jmir.1852

**Published:** 2012-06-27

**Authors:** Ricardo F Muñoz, Adrian Aguilera, Stephen M Schueller, Yan Leykin, Eliseo J Pérez-Stable

**Affiliations:** ^1^Internet World Health Research CenterDepartment of Psychiatry, San Francisco General HospitalUniversity of California, San FranciscoSan Francisco, CAUnited States; ^2^School of Social WelfareUniversity of California, BerkeleyBerkeley, CAUnited States; ^3^Department of PsychiatryUniversity of California, San FranciscoSan Francisco, CAUnited States

**Keywords:** Internet intervention, smoking cessation, international resources, Spanish, English, outcome study

## Abstract

**Background:**

Internet interventions have the potential to address many of the health problems that produce the greatest global burden of disease. We present a study illustrating this potential. The Spanish/English San Francisco Stop Smoking Internet site, which yielded quit rates of 20% or more at 12 months in published randomized controlled trials (RCTs), was modified to make it accessible to Spanish- and English-speaking smokers 18 years of age or older anywhere in the world.

**Objective:**

To illustrate that Internet interventions designed to conduct RCTs can be adapted to serve as universal health care resources. We also examine quit rates obtained in the current participant preference study (in which users could choose from all elements tested in previous RCTs) to determine whether they differ from the quit rates found in the RCTs.

**Methods:**

We modified the San Francisco Stop Smoking Internet site so that, instead of being randomly assigned to a specific intervention, participants could personalize the site by choosing among nine site elements (eg, stop smoking guide, reminder emails, journal, mood management intervention, or virtual group). Participants completed a baseline assessment, and reported smoking and mood data at 1-, 3-, 6-, and 12-month follow-ups. We assessed the modified website’s reach and outcomes (quit rates), and compared the quit rates of the current participant preference study with those of the previous RCTs.

**Results:**

In the first year of recruitment, 94,158 individuals from 152 countries and territories visited the site; 13,488 participants left some data; 9173 signed consent; 7763 completed the baseline survey; and 1955, 1362, 1106, and 1096 left 1-, 3-, 6-, and 12-month data, respectively. Observed quit rates were 38.1% (n = 668), 44.9% (n = 546), 43.6% (n = 431), and 45.4% (n = 449), respectively. The current participant preference study yielded higher observed quit rates (odds ratio 1.30) than the previous RCT when controlling for individuals’ demographic and smoking characteristics.

**Conclusions:**

After strict RCTs are completed, Internet intervention sites can be made into worldwide health intervention resources without reducing their effectiveness.

**Trial Registration:**

Clinicaltrials.gov NCT00721786; http://clinicaltrials.gov/ct2/show/NCT00721786 (Archived by WebCite at http://www.webcitation.org/66npiZF4y)

## Introduction

The concept of health care as a basic human right has been advanced by many. Franklin Roosevelt, in his 1944 message to Congress, called for “a second Bill of Rights” that would include “the right to adequate medical care and the opportunity to achieve and enjoy good health” [1]. This goal was included in the United Nations Universal Declaration of Human Rights [2]. However, the concept remains an aspiration that has not been actualized in most of the world. More than 40 years ago, Dr Martin Luther King Jr spoke to the Medical Committee for Human Rights, saying, “Of all the forms of inequality, injustice in health care is the most shocking and inhumane” [3]. We argue that self-help, automated Internet interventions could contribute to making the goal of adequate health care for all a reality. We illustrate this potential by describing how an Internet stop smoking intervention found effective in randomized controlled trials (RCTs) was made available freely to individuals worldwide.

In 2008, the World Health Organization released a report that stated that “Tobacco is the single most preventable cause of death in the world today...Unless urgent action is taken tobacco could kill *one billion people *during this century” [4]. To reduce this figure, smoking cessation interventions must be disseminated widely. There are over one billion smokers in the world today, 80% of whom reside in low- and middle-income countries [5]. Most communities do not offer smoking cessation interventions.

Internet interventions can fill a void in health care by providing evidence-based smoking cessation anytime, anywhere, to anyone with Internet access. Consumable interventions, in which the active agent is spent when administered, are expensive in terms of money, time, or both, so they are rarely used on a large scale. For example, smoking cessation groups consume the counselor’s time: each hour spent can never be used to serve another smoker. Nicotine patches or other pharmacological interventions can be used only once. Thus, each unit of time or money benefits only one individual or group of individuals. This makes such services expensive and thus difficult to establish and sustain. Nonconsumable interventions, such as automated self-help Internet interventions, are a great deal less expensive per user when delivered on a large scale. Though they require a modest amount of funding to host and maintain the website, after the interventions are provided to several thousand individuals (which is very feasible via the Web), the marginal cost (the cost of providing the same services to one more individual) approaches zero. This makes Internet interventions particularly useful if we want to blanket the world with evidence-based health interventions.

The San Francisco Stop Smoking Internet site was developed and tested in RCTs with funding from the California Tobacco-Related Disease Research Program, obtaining quit rates of 20% or more at 12 months [6,7], which are comparable with quit rates of 14%–27% reported for smoking cessation therapies or smoking cessation groups [7]. Our Internet interventions were extensions of a research program focused on Spanish-speaking smokers [8,9] and were translated and adapted to English to reach a larger number of smokers worldwide. After the grant funding ended for the last RCT, we decided not to close down the site, as would be done with most research grant-funded face-to-face interventions, but rather to keep it active and available as a service to smokers worldwide. We removed the randomization function and most eligibility criteria (except being 18+ years old), and allowed participants to choose any site element(s), thus conducting a participant preference study. These three changes allowed us to assess the dissemination and effectiveness of the intervention on a large scale and to experiment with a working model for how Internet research sites can be made accessible as basic services to any adult in the world with Internet access. This is a clear advantage of Internet interventions over traditional face-to-face outcome studies. When face-to-face trials end, even if they are highly successful, the investigators are almost always unable to continue to provide the same level of service to the public (mainly because staffing costs are prohibitive), leaving them to hope that the knowledge they have gathered will eventually be shared via adoption of their intervention by others. Internet interventions, such as the one described here, can reduce the average length of time that empirically supported interventions take to go from the laboratory to routine use. The US Institute of Medicine reported that it takes an average of 17 years for knowledge from RCTs to be made available to the public [10]. The San Francisco Stop Smoking site was switched from an RCT to an open participant preference site on the day the RCT ended.

This report is the fourth in a line of outcome papers based on the San Francisco Stop Smoking Internet website (www.stopsmoking.ucsf.edu in English; www.dejardefumar.ucsf.edu in Spanish). The first outcome paper [6] reported on the initial set of studies from the website, including single-condition studies and studies comparing two conditions at a time. The second outcome paper [7] compared four conditions with cohort maintenance efforts intended to reduce attrition at follow-up. These efforts included phone calls to the first 500 English-speaking and the first 500 Spanish-speaking participants. The four conditions were as follows: condition 1, The Stop Smoking Guide; condition 2, condition 1 + individually timed email messages; condition 3, condition 2 + a mood management intervention; and condition 4, condition 3 + a virtual group (an asynchronous bulletin board). After these first 1000 participants had been recruited, the site was left active and continued to recruit an additional 16,430 participants, who went through the randomized trial with the same four conditions in a totally automated manner, without the cohort maintenance efforts of the 2009 report [11].

In this paper, we report characteristics of the smokers and quit rates obtained from the sample recruited during the first year of the participant preference site. We compare these data to similar data obtained in the fully automated RCT with 16,430 participants. We hypothesized that providing choice would result in quit rates at least as high as those in the previous RCT.

## Methods

### Participants

Participants were recruited primarily through a Google Adwords campaign supported by a Google grant awarded to the senior researcher (RFM). The grant allowed us to bid up to US $1 to place sponsored links (ads) on pages resulting from Google searches relevant to smoking cessation. Individuals using search terms related to smoking were presented with a sponsored link to the English or Spanish version of the website (www.stopsmoking.ucsf.edu or www.dejardefumar.ucsf.edu). Spanish searches tended to have fewer advertisers competing for ad space; therefore, sponsored links for the site were more likely to appear on the first page of Google searches in that language. Others learned about the site through organic searches in Google and other search engines, links from other sites, word of mouth, and media sources.

### Study Procedures

The site offered all visitors to the site immediate access to the two major active intervention elements: the Stop Smoking Guide and the Nicotine Replacement Therapy Guide (see below for detailed description), which could be accessed from the home page without the need to sign up. Participants were informed that the site contained a program that is for smokers who are ready to quit; will take approximately 8 weeks to complete; and involves brief questionnaires at 1, 3, 6, and 12 months after enrollment to monitor progress with quitting and usage of the site. (This timing of follow-ups is common in cessation studies; it achieves a good balance between available data and low participant burden). Participants who clicked on a button to enroll in the study were directed to brief demographic and eligibility questionnaires. The sole eligibility criterion was being at least 18 years old. Those eligible and providing an email address received a password to log in to the site. This password also was used to sign the online consent form. Ineligible participants or those not consenting to participate in the research study could access the Stop Smoking Guide (which is itself an evidence-based intervention) and the Nicotine Replacement Therapy Guide from the home page of the website.

On logging in to the website, participants received a baseline questionnaire that assessed demographics, smoking patterns and history, and mood. After completing these assessments, participants were presented with the choice of nine intervention elements. Participants could select any and all of these elements. Unselected elements were suppressed. Participants were then taken to their individualized home page that contained a navigation bar with the elements they had selected. They could access these elements throughout the 12-month study. Participants received emails to complete follow-up assessments at 1, 3, 6, and 12 months after enrolling in the study. These intervals were identical to the ones used in our previous RCTs and thus are directly comparable.

### Intervention Elements

The participant preference version of the San Francisco Stop Smoking Internet site allowed users to choose among the following nine elements: (1) the Prequit Checklist, (2) The Stop Smoking Guide (*Guía*), (3) Nicotine Replacement Therapy Guide, (4) Taking Control of Your Life (*Tomando Control*), (5) individually timed email messages, (6) the Mood Management Intervention, (7) the Virtual Group, (8) the Journal, and (9) the Cigarette Counter.

#### The Prequit Checklist

This 10-item to-do list includes tips on removing smoking-related cues from one’s environment (eg, disposing of cigarettes, throwing out smoking supplies, and cleaning items that might smell of tobacco), identifying situations that might lead to relapses, and dealing with those situations.

#### The Stop Smoking Guide (Guía)

This is a National Cancer Institute evidence-based behavioral intervention originally developed for Spanish-speaking smokers [8,9,12], which provides empirical information about the effects of cigarettes, as well as methods for successful cessation.

#### Nicotine Replacement Therapy Guide

This guide outlines who should consider nicotine substitutes, information regarding options for nicotine substitutes (such as the nicotine patch, gum, and inhalers), and antidepressant medications used to help people stop smoking.

#### Taking Control of Your Life (Tomando Control)

This downloadable document helps individuals quit smoking by encouraging them to maintain a healthy mood state by engaging in pleasant, healthy activities [8,12]. The document instructs participants to keep a daily log of the number of positive activities, mood, and the number of cigarettes smoked. These materials were developed for an earlier study [9] and can be found on the National Cancer Institute’s site for Research Tested Intervention Programs [13].

#### Individually Timed Email Messages

These messages are brief tips and encouragement to stop smoking [14], keyed to the participant’s self-selected quit date.

#### The Mood Management Intervention

This 8-lesson cognitive–behavioral mood management course teaches the relationships between thoughts, activities, people, and one’s mood; it has been found effective at reducing symptoms of depression and increasing rates of quitting [9,15]. It includes tools to track mood, activities, thoughts, and interpersonal contacts, and to visualize how these are related to cigarettes smoked.

#### The Virtual Group

This asynchronous bulletin board provides an online forum where participants can post messages and respond to other users’ posts.

#### The Journal

A text box is provided for participants to keep notes on their progress toward their goals. Previous Journal entries can be viewed via a calendar. They can also be shared with the virtual group.

#### The Cigarette Counter

Participants indicate the number of cigarettes they smoked the previous day on a visual scale. Results from the cigarette counter are displayed graphically so participants can view their smoking patterns over time.

### Measures

A *demographic questionnaire *asked for age, gender, race/ethnicity, education, income, marital status.

A *smoking questionnaire *included age when the person started smoking, age when smoking regularly, number of cigarettes per day, confidence in quitting, smoking exposure, and smoking cessation methods used.

The *Fagerström Test for Nicotine Dependence *(FTND) [16] is a commonly used 6-item test of nicotine dependence, with a range from 0 to 10.

The *Major Depressive Episode (MDE) Screener *(Mood Screener) [17] screens for the presence of the 9 symptoms of current and past MDEs according to the *Diagnostic and Statistical Manual of Mental Disorders *(DSM-IV-TR) [18], as well as for criterion C (significant impairment in functioning). This instrument has been shown to have good agreement with the Primary Care Evaluation of Mental Disorders [19,20] and with clinician-administered interviews [21].

The *Center for Epidemiologic Studies Depression *scale (CES-D) [22] is a 20-item self-report scale designed to measure the current level of depressive symptoms.

*Self-reported 7-day point prevalence abstinence *was defined as a “no” response to “Have you smoked 1 or more cigarettes in the last 7 days?” at 1-, 3-, 6-, and 12-month follow-ups. This is the main measure used in the previously published RCT [7].

### Statistical Analyses

We constructed two logistic regression models to predict quit rates. Predictors were age, sex, education, language, FTND score, number of cigarettes smoked per day, age when the person started smoking, number of quit attempts, use of quit aids, CES-D score, and presence of lifetime MDEs. Variables were entered stepwise in groups: demographic variables, smoking variables, and depression variables. One model assessed differences between all participants in the current participant preference study and the previous RCT. Another model tested differences only among individuals who would have met the more-stringent requirements of the previous RCT (smoked more than 5 cigarettes a day, intending to quit in the next month, used email at least once weekly, and was 18+ years of age). Chi-square and independent sample *t *tests were also conducted with descriptive data to make comparisons as in the case of attrition data, as well as to compare between the Spanish- and the English-speaking subsamples.

Because the website was open to anyone 18 years of age or older, some participants were nonsmokers at baseline. We analyzed outcome excluding these nonsmokers.

## Results

### Sample

**Figure 1:**
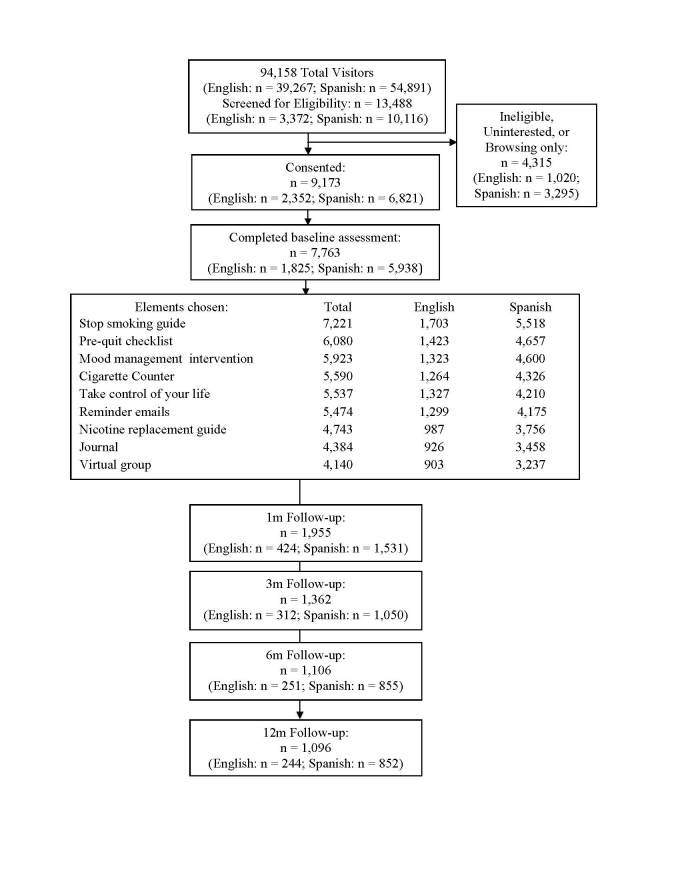
Progression of participants through the San Francisco Stop Smoking participant preference site, with follow-up at 1 month (1m), 3 months (3m), 6 months (6m), and 12 months (12m).

As seen in Figure 1, during a 1-year period (September 19, 2008 through September 18, 2009), 94,158 (n = 39,267 English-speaking, n = 54,891 Spanish-speaking) individuals from 152 countries and territories visited the site; 13,488 (n = 3372 English-speaking, n = 10,116 Spanish-speaking) participants left data; 9173 (n = 2352 English-speaking, n = 6821 Spanish-speaking) signed consent; and 7763 (n = 1825 English-speaking; n = 5938 Spanish-speaking) participants completed a baseline survey. Participants completing baseline represented 124 countries and territories. English speakers came from 111 countries, of which the top 3 were India (n = 442), the United States (n = 240), and South Africa (n = 182). Spanish speakers came from 51 countries, of which the top 3 were Spain (n = 2573), Argentina (n = 1114), and Mexico (n = 547). Table 1 further describes sample characteristics. Table 2 describes the smoking and depression characteristics of participants who identified as smokers or reported smoking 1+ cigarettes at baseline (n = 7353). As can be seen from Table 2, Spanish-language participants appeared to be less addicted to nicotine; they were also far less likely to report using quit aids in the past 6 months and more likely to screen positive for current MDE.

**Table 1:** Demographics of consenting participants who completed baseline assessment.

Characteristic		Smokers			Nonsmokers
		All (n = 7353)	English-speaking (n = 1693)	Spanish-speaking (n = 5660)	All (n = 410)
**Sex** ^a^		n = 7314	n = 1688	n = 5626	n = 410
	Men	4024 (55.02%)	1099 (65.11%)	2925 (51.99%)	208 (50.7%)
	Women	3290 (44.98%)	589 (34.9%)	2701 (48.01%)	202 (49.3%)
**Ethnicity** ^a,b^		n = 7286	n = 1658	n = 5628	n = 406
	Hispanic/Latino	5538 (76.01%)	185 (11.2%)	5232 (92.96)	273 (67.2%)
	Not Hispanic/Latino	1752(23.99%)	1356 (81.79%)	396 (7.04%)	133 (32.8%)

**Race ^a,c^**		n = 7097	n = 1478	n = 5619	n = 407
	European descent	4435 (62.49%)	617 (41.7%)	3818 (67.95%)	280 (70.3%)
	Asian descent	531 (7.5%)	520 (35.2%)	11 (0.2%)	19 (5%)
	African descent	48 (1%)	42 (3%)	6 (0.1%)	2 (1%)
	Indigenous descent	20 (0.3%)	3 (0.2%)	17 (0.3%)	0 (0%)
	Other/multiethnic	1292 (18.20%)	253 (17.1%)	1019 (18.13%)	76 (18.6%)
	Mestizo	771 (10.9%)	23 (2%)	748 (13.3%)	23 (5.7%)
**Education** ^a,b^		n = 7216	n = 1664	n = 5552	n = 406
	High school or less	1688 (23.39%)	296 (17.8%)	1392 (25.07%)	62 (15%)
	Some college	2626 (36.39%)	428 (25.7%)	2198 (39.59%)	129 (31.8%)
	College graduate	2126 (29.46%)	622 (37.4%)	1504 (27.09%)	139 (34.2%)
	Graduate degree	776 (10.75%)	318 (19.1%)	458 (8.2%)	76 (18%)
**Marital status** ^a,d^		n = 7345	n = 1690	n = 5655	n = 410
	Single	2647 (36.04%)	722 (42.7%)	1925 (34.04%)	114 (28.0%)
	Cohabiting	1224 (16.66%)	195 (11.5%)	1029 (18.20%)	83 (20.2%)
	Married	2674 (36.41%)	626 (37.0%)	2046 (36.18%)	167 (40.7%)
	Separated	354 (4.8%)	55 (3%)	299 (5.3%)	16 (4%)
	Divorced	395 (5.4%)	82 (5%)	313 (5.5%)	25 (6%)
	Widowed	53 (1%)	10 (1%)	43 (1%)	4 (1%)

^a ^Difference between English- and Spanish-speaking participants is significant at *P *< .001 level.

^b ^Difference between smokers and nonsmokers is significant at *P *< .001 level.

^c ^Difference between smokers and nonsmokers is significant at *P *= .003 level.

^d ^Difference between smokers and nonsmokers is significant at *P *= .03 level.

**Table 2:** Smoking and clinical characteristics of consenting smokers.

Characteristic			All (n = 7353)	English-speaking (n = 1693)	Spanish-speaking (n = 5660)	*P *value (English vs Spanish)
**Smoking history, mean (SD)**						
	Age (years)		35.0 (10.7)	33.6 (10.7)	35.8 (10.7)	<.001
	Age (years), first cigarette		15.8 (3.3)	16.6 (4.1)	15.5 (3.0)	<.001
	Age(years) regular smoker		18.8 (4.4)	19.0 (4.3)	18.8 (4.4)	.07
	Cigarettes per day		18.1 (10.0)	16.7 (10.0)	18.5 (10.0)	<.001
	FTND^a ^score		6.5 (2.6)	7.4 (2.0)	6.2 (2.7)	<.001
	Quit confidence		6.6 (2.3)	6.7 (2.3)	6.6 (2.3)	.04
**Methods used to quit in last 6 months, n (%)**						
	Nicotine gum		884 (12.0%)	235 (13.9%)	649 (11.5%)	0.008
	Nicotine patch		580 (7.9%)	220 (13.0%)	360 (6.4%)	<.001
	Nicotine inhaler		60 (1%)	41 (2%)	19 (0.3%)	<.001
	Nicotine spray		18 (0.2%)	10 (1%)	8 (0.1%)	.003
	Bupropion		223 (3.0%)	64 (4%)	159 (2.8%)	.04
	Varenicline		170 (2.3%)	37 (2%)	133 (2.3%)	.78
	Other antidepressant		103 (1.4%)	29 (2%)	74 (1%)	.24
	Stop smoking group		124 (1.7%)	27 (2%)	97 (2%)	.83
	Hypnosis		116 (1.6%)	56 (3%)	60 (1%)	<.001
	Acupuncture		126 (1.7%)	29 (2%)	97 (2%)	1.00
	Motivational tapes		126 (1.7%)	57 (3%)	69 (1%)	<.001
	Other self-help		321 (4.4%)	118 (7.0%)	203 (3.6%)	<.001
	Other websites		188 (2.6%)	51 (3%)	137 (2.4%)	.19
	Prayer		271 (3.7%)	119 (7.0%)	152 (2.7%)	<.001
	Consultation with doctor		239 (3.3%)	65 (4%)	174 (3.1%)	.12
	Other		328 (4.5%)	108 (6.4%)	220 (3.9%)	<.001
	None		5047 (68.64%)	997 (58.9%)	4050 (71.55%)	<.001
**Depression variable, n (%)**						
	Major depressive episodes					
		No history	4968 (67.56%)	1143 (67.51%)	3825 (67.58%)	.80
		Past only	1027 (13.97%)	283 (16.7%)	744 (13.1%)	<.001
		Current	1358 (18.47%)	267 (15.8)%	1091 (19.3%)	.002
	CES-D^b ^score, mean (SD)		17.7 (12.2)	17.9 (11.9)	17.7 (12.3)	.35

^a ^Fagerström Test for Nicotine Dependence.

^b ^Center for Epidemiologic Studies Depression scale.

**Table 3:** Self-reported 7-day smoking abstinence, for all smokers and, separately, for smokers eligible for the previous RCT^a ^(% quit).

			All (n = 7353)	English-speaking (n = 1693)	Spanish-speaking (n = 5660)	*P *value (English vs Spanish)
**Observed quit rate**						
	All smokers					
		Month 1	668 (38.1%)	141 (38.8%)	527 (37.9%)	.77
		Month 3	546 (44.9%)	115 (42.9%)	431 (45.5%)	.49
		Month 6	431 (43.6%)	100 (47.8%)	331 (42.5%)	.19
		Month 12	449 (45.4%)	104 (50.0%)	345 (44.2%)	.14
	RCT eligible					
		Month 1	587 (40.4%)	127 (40.1%)	460 (40.5%)	.90
		Month 3	466 (45.4%)	101 (43.0%)	365 (46.1%)	.42
		Month 6	378 (45.9%)	87 (49%)	291 (45.0%)	.35
		Month 12	377 (45.8%)	93 (51%)	284 (44.2%)	.11
**Missing = smoking** ^b^						
	All smokers					
		Month 1	668 (9.1%)	141 (8.3%)	527 (9.3%)	.23
		Month 3	546 (7.4%)	115 (6.8%)	431 (7.6%)	.27
		Month 6	431 (5.9%)	100 (5.9%)	331 (5.8%)	.96
		Month 12	449 (6.1%)	104 (6.1%)	345 (6.1%)	.96
	RCT eligible					
		Month 1	587 (9.8%)	127 (8.8%)	460 (10.1%)	.17
		Month 3	466 (7.8%)	101 (7.0%)	365 (8.0%)	.24
		Month 6	378 (6.3%)	87 (6%)	291 (6.4%)	.71
		Month 12	377 (6.3%)	93 (7%)	284 (6.2%)	.76

^a ^Smokers who met eligibility criteria set for the 2009 randomized controlled trial (RCT) [7,11].

^b ^Assuming that every participant not reporting data is still smoking.

### Attrition

Of the 7763 consenting participants who completed the baseline survey, 1955 (25.18%), 1362 (17.54%), 1106 (14.25%), and 1096 (14.12%) left 1-, 3-, 6-, and 12-month data, respectively. Most participants (4914, 63.30%) did not return to the site for any of the follow-ups. Of the 2855 (36.78%) who left follow-up data, 1423 (49.84%) responded to one, 629 (22.0%) responded to two, 367 (12.8%) responded to three, and 436 (15.3%) responded to all four follow-ups. These rates were numerically lower than those obtained in the RCT. We found no differences in the proportion of follow-ups completed by English or Spanish speakers (χ^2^_4 _= 6.38, *P *= .17). There was a significant difference in the proportion of completed follow-ups between women and men (χ^2^_4 _= 37.88, *P *< .001), with the largest difference between those who completed no follow-ups (2798, 66.12% men vs 2105, 60.28% women).

### Quit Rates (7-Day Abstinence)

In Table 3, we report both the observed quit rates (based on those reporting data at each follow-up) and the missing = smoking quit rates (that is, assuming that every participant not reporting data is still smoking) for participants in the participant preference trial. We ran a logistic regression model to compare the observed quit rates between the previous RCT and the current participant preference study controlling for demographic, smoking, and depression variables. We found that participants in the current participant preference study reported higher quit rates than participants in the previous RCT (Wald χ^2^_1 _= 10.43, *P *< .001, odds ratio [OR] 1.30). With both samples combined, those with more education were more likely to quit (Wald χ^2^_1 _= 14.51, *P *< .001, OR 1.14); English-speaking participants were less likely to do so (Wald χ^2^_1 _= 6.52, *P *= .01, OR 0.81). The only smoking-related variable related to quit rates was nicotine dependence, such that higher dependence corresponded to lower quit rates (Wald χ^2^_1 _= 13.30, *P *< .001, OR 0.94). Both levels of depressive symptoms (Wald χ^2^_1 _= 20.80, *P *< .001, OR 0.99) and history of MDEs (Wald χ^2^_1 _= 8.69, *P *= .003, OR 0.79) predicted lower quit rates in participants. The largest change in *R* *2 *resulted from the addition of the depression variables.

**Figure 2:**
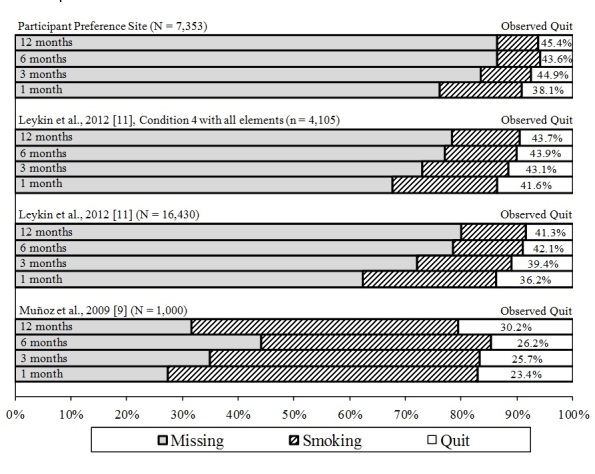
Observed quit rates by study for the San Francisco Stop Smoking participant preference study and previous randomized controlled trials (RCTs). The outcomes of the current participant preference site, as compared with the RCT with live cohort maintenance follow-up methods ([7], N = 1000), a fully automated RCT without live follow-up ([11], N = 16,430), and condition 4, the condition in that same automated RCT that included all site elements, which approximates the current site, but without the participant preference component ([11], n = 4105). Shaded areas indicate missing data. Observed Quit is defined as the number reporting not smoking divided by number reporting smoking status (smoking or not smoking). Thus, it does not impute values for missing data.

Figure 2 displays the quit rates obtained from this study along with those obtained in previous RCTs. The observed 1-, 3-, 6-, and 12-month quit rates among smokers in the current study were 38.1%, 44.9%, 43.6%, and 45.4%, respectively. Restricting the sample only to participants who met eligibility criteria for the most recent, fully automated RCT [11] produced observed quit rates of 40.4% (n=587), 45.4% (n=378), 45.9% (n=378), and 45.8% (n=377) respectively. In that RCT, quit rates at the same time points for participants in condition 4, who received all elements (the most reasonable comparison), were 41.6% (552/1328), 43.1% (475/1103), 43.9% (414/943), and 43.7% (386/884). Note that in the 2009 RCT, we instituted cohort maintenance methods to obtain as complete a set of follow-up data as possible by phoning participants who did not respond to automated emails. This resulted in follow-up rates as high as 69% at 12 months. The fully automated RCT reported in the Leykin et al [11] and the participant preference site results did not include these phone calls. Thus, the observed quit rates are based on a much smaller proportion of respondents. This needs to be taken into account when interpreting these figures.

## Discussion

This study illustrates that it is feasible to blanket the world with self-help automated Internet interventions for major global health problems. We showed that turning an efficacious Internet-based RCT into a participant preference study site immediately after the RCT ended resulted in quit rates similar to or better than those found in the RCT. Opening up an Internet intervention research site after completion of an Internet outcome study allows people worldwide to continue to use it, and the outcomes can be similar to those obtained within a strict RCT context.

We found higher observed quit rates among those in the participant preference study. In addition, several variables predicted higher levels of quit rates. Specifically, higher levels of education, visiting the Spanish version of the site, lower levels of nicotine dependence, lower depressive symptoms, and no history of MDEs were all more highly associated with increased quit rates. We found the higher quit rates related to the Spanish site intriguing. Perhaps because Spanish-speaking smokers were less likely to have used other smoking cessation resources (see Table 2), the Internet site was more likely to be of help to them in part by helping those who had an easier time quitting. English speakers able to quit more easily may have already quit using other methods. This hypothesis is supported by the fact that English speakers had significantly higher FTND nicotine dependence scores (see Table 2). If this is correct, it is one more argument for blanketing the world with evidence-based Internet interventions, so that individuals in locations that have no health resources can have access to at least a basic level of care, which can be effective in addressing the targeted health problem. Note, in Table 2, that 2 out of 3 smokers had used nothing prior to our Internet intervention. Only 3.3% had consulted with a doctor, and 1.7% had attended a smoking cessation group. The most used aid was nicotine gum, but even that method was used by only 12% of smokers.

These findings illustrate how an automated stop smoking site can be used to provide a basic level of health care. Just as visits to professionals might help refer participants to additional or more-specialized services, an Internet stop smoking site can serve as a basic level of health care that could link participants to additional services if the basic level is not enough to deal with their health issues.

To help the reader consider the comparative advantage of this Internet intervention, we estimated the contribution of the site relative to comparable spending on alternative interventions. The San Francisco Stop Smoking site provided free access to the Stop Smoking Guide and Nicotine Replacement Therapy Guide to 94,158 visitors to the site. This would be comparable with providing that many printed brochures and mailing them to smokers all over the world. As with brochures, of course, there is no guarantee they would be read and acted upon. To serve the 9173 smokers who signed consent with other widely used interventions, it would have cost US $2,008,520 in nicotine patches (at $3.91 per day at 8 weeks per person), $2,984,527 in nicotine gum (at $5.81 per day and 8 weeks per person), $2,208,858 in bupropion (at $4.30 per day and 8 weeks per person) [23], and $1,174,144 in group counseling (at $16 per hour of counselor time once per week [24]. In comparison, the cost of maintaining the self-help, automated site for 1 year was approximately US $50,000 (hosting and maintenance costs, and the cost of a half-time bilingual staff person to handle email inquiries and check the site periodically).

To have the greatest impact in reducing unnecessary suffering due to health problems, we recommend that international agencies and Internet intervention researchers prioritize (1) the health problems producing the greatest burden of disease globally, such as smoking, which is the number one cause of preventable death, and depression, which is the number one cause of disability worldwide [25], (2) the health problems that can be addressed using behavior change interventions, and (3) the languages that reach the largest number of people via the Web (currently English, Chinese, and Spanish). At the same time, we want to avoid duplicating the problem of orphan drugs, which are neglected because they work for less-common disorders. To do so, we encourage researchers with the expertise to address less-prevalent health issues and languages spoken by smaller populations to focus on these issues as well. For health care to be a right for all, it should include even numerically small minorities.

If we blanketed the world with automated, self-help Internet interventions such as the San Francisco Stop Smoking Internet site as a basic level of health care, many people without appropriate health care access could benefit from the online intervention. Communities with more resources would be able to add more-expensive and consumable services, in this case, additional smoking cessation aids. For example, they could provide smoking cessation groups that use the website as an adjunct, or they could provide nicotine patches, gum, and other pharmacological aids to smokers using the website. These communities might also learn how to better link basic, scalable levels of health care, such as Internet tools, to more-involved forms of care. The stop smoking site might have served as a portal for identifying those interested and motivated to quit, providing basic skills that apparently benefitted many, and providing motivation for further care for those who need it. Linked resources could help alleviate concerns that those who visit Internet sites and do not respond will feel discouraged from seeking out future support, especially if they are explicitly told that this is something that will help many, but not all, and that some will need more-individualized treatment.

We have presented a successful example of developing an Internet health intervention, testing it in strict RCTs, and immediately disseminating it to a large worldwide population. We suggest that international health institutions, such as the World Health Organization, establish official, adequately funded programs to systematically develop, test, and disseminate evidence-based, self-help, automated, and multilingual interventions to all who need them. This will contribute to the goal of making health care a freely accessible right for people worldwide.

## Abbreviations

CES-D: Center for Epidemiologic Studies Depression scale
FTND: Fagerström Test for Nicotine Dependence
MDE: major depressive episode
OR: odds ratio
RCT: randomized controlled trial
